# Numerical model of a valvuloplasty balloon: in vitro validation in a rapid-prototyped phantom

**DOI:** 10.1186/s12938-016-0155-4

**Published:** 2016-04-12

**Authors:** Benedetta Biffi, Giorgia M. Bosi, Valentina Lintas, Rod Jones, Spyros Tzamtzis, Gaetano Burriesci, Francesco Migliavacca, Andrew M. Taylor, Silvia Schievano, Giovanni Biglino

**Affiliations:** Centre for Cardiovascular Imaging, UCL Institute of Cardiovascular Science & Great Ormond Street Hospital for Children, Great Ormond Street, London, WC1N 3JH UK; Department of Medical Physics and Biomedical Engineering, University College London, London, UK; Laboratory of Biological Structure Mechanics (LaBS), Chemistry, Materials and Chemical Engineering Department “Giulio Natta”, Politecnico di Milano, Milan, Italy; Department of Mechanical Engineering, UCL Faculty of Engineering Science, London, UK

**Keywords:** Valvuloplasty balloons, Percutaneous procedures, Validation of computational models, Implantation site material properties, 3D printing/rapid prototyping materials

## Abstract

**Background:**

Patient-specific simulations can provide insight into the mechanics of cardiovascular procedures. Amongst cardiovascular devices, non-compliant balloons are used in several minimally invasive procedures, such as balloon aortic valvuloplasty. Although these balloons are often included in the computer simulations of these procedures, validation of the balloon behaviour is often lacking. We therefore aim to create and validate a computational model of a valvuloplasty balloon.

**Methods:**

A finite element (FE) model of a valvuloplasty balloon (Edwards 9350BC23) was designed, including balloon geometry and material properties from tensile testing. Young’s Modulus and distensibility of different rapid prototyping (RP) rubber-like materials were evaluated to identify the most suitable compound to reproduce the mechanical properties of calcified arteries in which such balloons are likely to be employed clinically. A cylindrical, simplified implantation site was 3D printed using the selected material and the balloon was inflated inside it. The FE model of balloon inflation alone and its interaction with the cylinder were validated by comparison with experimental Pressure–Volume (*P*–*V*) and diameter–Volume (*d*–*V*) curves.

**Results:**

Root mean square errors (*RMSE*) of pressure and diameter were *RMSE*_*P*_ = 161.98 mmHg (3.8 % of the maximum pressure) and *RMSE*_*d*_ = 0.12 mm (<0.5 mm, within the acquisition system resolution) for the balloon alone, and *RMSE*_*P*_ = 94.87 mmHg (1.9 % of the maximum pressure) and *RMSE*_*d*_ = 0.49 mm for the balloon inflated inside the simplified implantation site, respectively.

**Conclusions:**

This validated computational model could be used to virtually simulate more realistic valvuloplasty interventions.

## Background

**Fig. 1:**
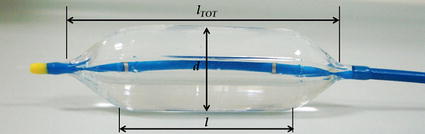
Edwards valvuloplasty balloon; *l*_*tot*_ is the total length of the balloon (75 mm), *l* is the cylindrical portion (40 mm length) and *d* is the diameter (23 mm)

Balloons are cardiovascular devices adopted in several minimally invasive procedures, such as valvuloplasty [1, 2], sizing [3], aortic coarctation expansion [4], angioplasty [5], stent or percutaneous valves [6] expansion. In such procedures, a catheter is inserted in a vessel via percutaneous access under fluoroscopy guidance, and the deflated balloon is positioned at the target site where is then subsequently inflated with contrast medium [7]. Balloons are generally characterised by the following parameters: nominal and burst pressure, nominal diameter *(*i.e. the diameter at which the nominal pressure is reached), and balloon length—typically referred as the length of the cylindrical region (Fig. 1). Depending on their working pressure and material properties, they are often categorised as “compliant” and “non-compliant” balloons. The latter work at high pressure (≫1 atm) and are made of non-compliant materials, such as PET and Nylon, which exhibit high tensile strength with relatively low elongation, maintaining their designed size and shape even at high pressures. Due to these properties, they are adopted in procedures meant to impose large deformations (e.g., to anatomical structures or to a device). During balloon aortic valvuloplasty, for instance, a non-compliant balloon is inflated across the aortic valve annulus to induce the separation of the leaflet commissures and/or fracture the calcifications [8], thus increasing the aortic valve effective orifice area and dropping the mean pressure gradient [9]. Despite short-term positive outcomes, the survival rates of long-term follow-up patients are low, and comparable to the natural course of untreated patients [10]. Although their limited success as a therapeutic option, the number of performed BAV has increased significantly with the advent of transcatheter aortic valve replacement (TAVR), where the balloon is used to pre-dilate the stenosed valve and facilitate TAVR delivery, or as a bridging therapy [9, 10].

Computational analysis simulating surgical or minimally invasive procedures of cardiovascular devices implantation have been extensively developed, demonstrating to be a useful tool to study procedures feasibility and to understand potential complications [11]. Within cardiovascular devices, also non-compliant balloons have been modelled in several numerical works, both as part of studies on stent expansion procedures [12–17] and as angioplasty balloons studies per-se [18–21]. However, a thorough validation of balloon behaviour with specifically acquired experimental data is lacking.

Therefore, the aim of this work is to present a novel methodology to validate a finite element (FE) model of a non-compliant valvuloplasty balloon interacting with a realistic implantation site. It has been shown [12] that a correct understanding of the tissue response requires a faithful reproduction of the real balloon mechanical behaviour in terms of the mutually dependent variables i.e., volume, pressure and diameter. In this work, different rapid prototyping materials are evaluated in order to identify a suitable compound to replicate the mechanical properties of implantation sites in which valvuloplasty balloons are clinically used. Also, experimental data of balloon material and balloon inflation characteristic curves are collected, in order to quantitatively describe both free and constrained inflation, in terms of volume, pressure and diameter. Ultimately, we hypothesize that FE models can satisfactorily recapitulate isolated balloon behaviour and the balloon’s interaction with a 3D printed implantation site of realistic mechanical properties, thus validating the numerical model against purposely acquired experimental data.

## Methods

A non-compliant valvuloplasty balloon (Edwards 9350BC23, Edwards Lifesciences LLC, Irvine, CA, USA) with the following characteristics was chosen for this study: nominal diameter = 23 mm, cylindrical region length = 40 mm, length = 75 mm, nominal volume = 21 ml, nominal pressure = 4 atm, burst pressure = 6 atm.

## Experimental methods

### Balloon characterisation

In order to characterise the valvuloplasty balloon, two approaches were undertaken. Firstly, the balloon material was derived from tensile testing, and then the balloon behaviour in terms of Pressure–Volume (*P*–*V*) and diameter–Volume (*d*–*V*) curves was obtained in free inflation experiments.

To obtain the uniaxial stress–strain curve of the balloon membrane, two longitudinal and circumferential specimens were tested to failure, following the standards BS-ISO-27:2005 (Roellr Z5.0, Zwick GmbH & Co.,Ulm, Germany). Stress–strain (σ-ε) data were analysed to assess isotropy within the range of deformations experienced by the balloon during inflation (ε < 10 %). In order to appreciate and evaluate the elastoplastic effect typical of rubber-like materials, one circumferential dumb-bell specimen was tested under cyclic conditions. Ten loading and unloading cycles were performed at a maximum strain of 10 %.

**Fig. 2:**
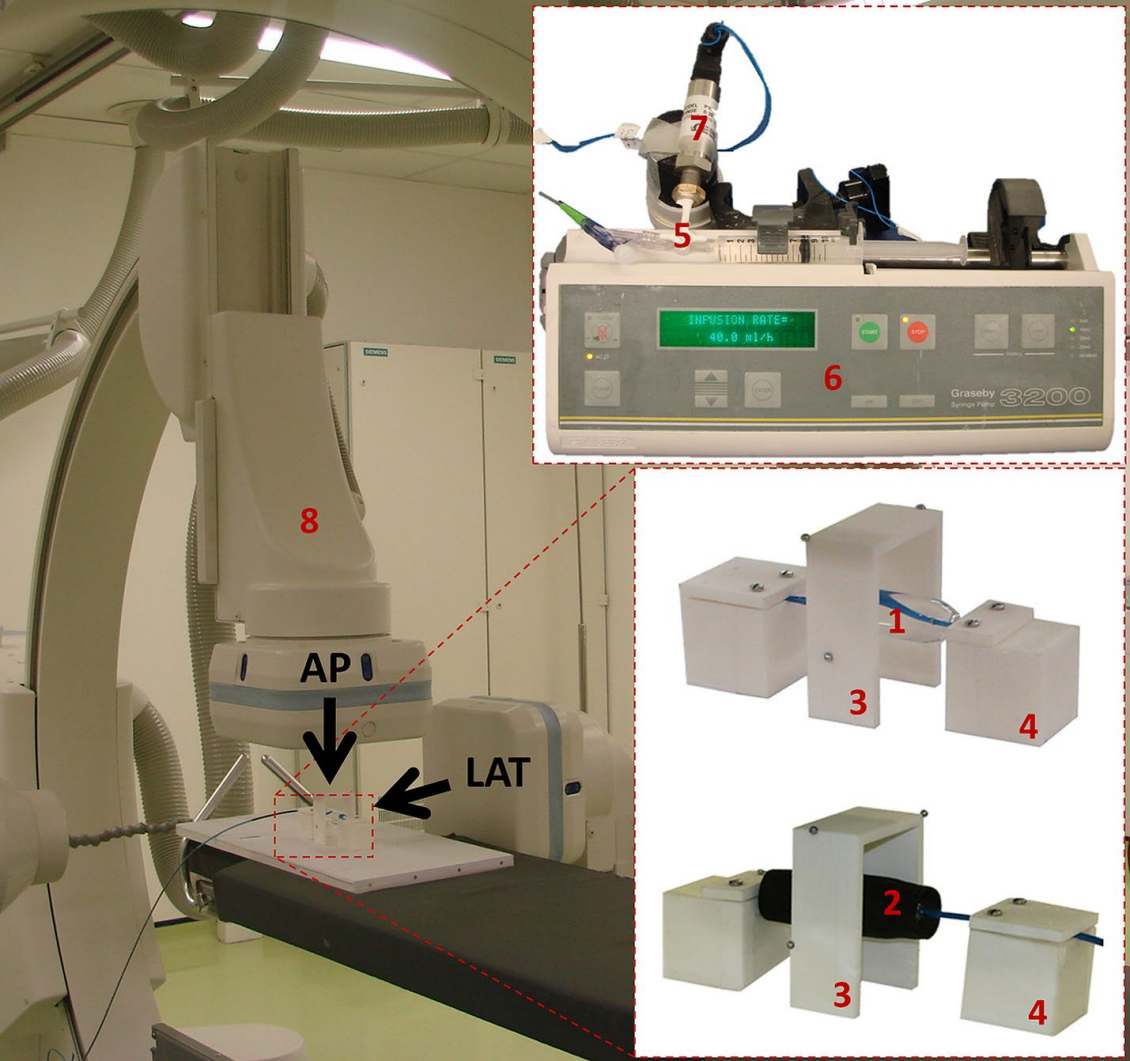
Experimental set-up for inflation test on balloon: (*1*) balloon in the free inflation, (*2*) balloon inside simplified anatomical site, (*3*) arch for fluoroscopy images calibration, (*4*) balloon holders, (*5*) 3-way valve, (*6*) syringe pump, (*7*) pressure transducer, (*8*) biplane fluoroscopy scanner: *AP* antero-posterior projection, *LAT* lateral projection

Bench tests of balloon free inflation were carried out with the experimental set-up shown in Fig. 2. Two holders were purposely designed and 3D printed in order to suspend and constrain the balloon during the inflation experiment. The balloon inflation was controlled by a syringe pump (Graseby^®^ 3200, Smiths Group, London, UK) equipped with a 10 ml syringe. Pressure was measured with a pre-calibrated strain gauge pressure transducer (PXM-4101 Omega Eng. Inc., Stamford, CT, USA, full scale range = 0 ÷ 10 bar) connected to the balloon catheter by means of a 3-way valve. In order to gather information about balloon geometry over time, the whole experiment was performed under biplane fluoroscopy (AXIOM Artis; Siemens, Erlangen, Germany). X-ray images (isotropic image resolution = 0.5 mm/pixel) from orthogonal antero-posterior (AP) and lateral (LAT) projections of the balloons were acquired during inflation in order to reconstruct the balloon contours over time. An arch-shaped component was included in the set-up for fluoroscopy image calibration [22]. In particular, four metallic beads (0.5 mm diameter) were glued to the arch at known positions, providing high contrast landmarks of reference distances for calibration purposes.

Each experiment was carried out as follows. The balloon was filled with diluted contrast medium (1:1 volume) until it reached the maximum volume corresponding to zero pressure, and then inflated with a constant flow rate (40 ml/h) up to the end of the syringe stroke. *P* was continuously recorded and fluoroscopy images taken with a sampling rate of 1 image every 15 s. Measurements were performed in air and repeated three times using the same balloon.

Fluoroscopy images were post-processed with a semi-automatic method herein developed, allowing the extraction of *V* and *d* at every frame. A calibration coefficient was computed for each AP and LAT projection as:

$$w = \frac{{b_{1,real} }}{{2 \cdot b_{1,image} }} + \frac{{b_{2,real} }}{{2 \cdot b_{2,image} }}$$

where *b*_1,*real*_ and *b*_2,*real*_ are the known projected distances between the metallic beads, and *b*_1,*image*_ and *b*_2,*image*_ are the corresponding measures on the image. Two values of distance were measured on each image and then averaged, in order to minimise the error due to X-rays beam distortion. The balloon was segmented on each AP and LAT projection by applying a thresholding filter on the greyscale 2D images (Mimics, Materialise, Leuven, Belgium), and the balloon contour was extracted as a polyline. Polylines were then imported in a CAD software (Rhinoceros, McNeel & Associates, USA) and scaled with the corresponding calibration coefficient. Each contour was revolved around the balloon longitudinal axis and *V*_*0*_ was obtained by averaging the volumes of the solid at the initial frame from the AP and LAT projections. Balloon volume change during the inflation was computed as $V\left( t \right) = V_{0} + \dot{V} \cdot \Delta T,$ where $\dot{V}$ is the flow rate imposed by the syringe pump (40 ml/h) and $\Delta T$ is the time increment. Balloon diameter was measured from all images as the distance between the balloon contours in the central section. The averaged values of AP and LAT diameters were used to derive the *d*–*V* curves, and *P* and *V* data were used to derive the *P*–*V* curves.

### Rapid prototyping material characterisation

Compounds obtained from mixing different percentages of commercially available rapid prototyping (RP) materials TangoPlus FullCure^®^930 and VeroWhite FullCure^®^830 were characterised to identify materials suitable for replicating the mechanical properties of arteries. The former is a rubber-like flexible material, while the latter is more rigid and robust, offering dimensional stability. The considered compounds combinations are reported in Table 1.

**Table 1:** Composition and name of the adopted RP composites

Composite name	% TangoPlus	% VeroWhite
T100	100	0
T92	92	8
T83	83	17
T75	75	25
T50	50	50

Cylindrical samples were printed with PolyJet™ (3D printer Objet Connex500, Stratasys Ltd. ©), a RP technology which deposits photopolymer materials adopting a layer by layer approach (layer thickness 16 μm). Layers were deposited “vertically”, i.e., each cylinder was built from the bottom to the top, growing along its longitudinal dimension.

Compliance tests as described in [23] were performed on RP cylindrical samples to assess the effect of varying material composition and thickness on the distensibility (*D*), defined as

$$D = {{\Delta V} \mathord{\left/ {\vphantom {{\Delta V} {\Delta P \cdot V_{in} }}} \right. \kern-0pt} {\Delta P \cdot V_{in} }}$$

with *V*_*in*_ = initial volume. In particular, four cylinders (inner diameter, *d* = 20 mm, length, *l* = 60 mm, thickness, *t* = 0.8 mm) were printed in T100, T92, T83, T75, and three cylinders (*d* = 20 mm, *l* = 60 mm, and *t* = 0.8, 1.0 and 1.2 mm) were printed in T100. Thicknesses were selected according to previous work [24]. A thickness of 0.6 mm was the minimum allowed by the printing technology to ensure integrity of the printed parts.

Assuming the samples to behave like thin-walled cylinders of elastic, isotropic and homogeneous material, the Young’s Modulus (*E*) was derived from the Laplace’s law as:

$$E = \sigma_{\vartheta } /\varepsilon$$

$$\sigma_{\vartheta } = P \cdot r/t$$

$$\varepsilon = (r - r_{in} )/r_{in}$$

$$r = \sqrt {V/\pi \cdot l}$$

Based on the evaluation of RP materials properties, T50 was deemed the most suitable material to reproduce the mechanical property of a calcified aorta (*D* as reported in [24] and *E* = 13.4 ÷ 31.8 MPa in patients [25, 26]). Properties of the chosen material are further discussed in the “Experimental results” section. Compliance test on a cylindrical sample (*d* = 14.6 mm, *t* = 1.2 mm, *l* = 60 mm) printed in T50 was performed to derive *D* of the selected material and thickness combination. Uniaxial tensile test on dumb-bell samples with width = 2 mm, thickness = 1 mm, length = 25 mm were performed as described for the balloon material, and *E* was computed assuming linear elastic isotropic behaviour. This assumption was made in order to compare the RP material properties with the available literature data [25, 26] which describe the mechanical properties of calcified arteries in terms of Young’s Modulus.

### Inflation test of balloon inside a simplified implantation site

A cylinder representing an idealised aorta was integrated in the experimental set-up adopted for the balloon alone. Considering the result of the RP materials evaluation, a 1.2 mm thick cylinder was manufactured with T50, as the most suitable compound to replicate a calcific aorta. The diameter of the cylinder (*d* = 22 mm) was selected considering the average value of aortic valve annulus that clinically would require the adoption of the considered balloon size. The length of the cylinder was set equal to that of the balloon (*l* = 75 mm).

The inflation test was repeated after positioning the valvuloplasty balloon inside the cylinder (Fig. 2), and *P*–*V* and *d*–*V* curves were obtained with the same method developed for the balloon alone. The same balloon was used to repeat the experiment three times.

## Computational methods

All simulations were performed with FE software Abaqus/Explicit (Dassault Systèmes Simulia, Providence, RI, US) considering quasi-static conditions.

### Validation of balloon FE model

The shape of the valvuloplasty balloon is displayed in Fig. 1, showing a central cylindrical region closed by two pseudo-conical heads. The balloon model, designed from the contour extracted from the fluoroscopy images and with uniform membrane thickness (0.09 mm), was discretised with not-reduced membrane elements (6426 elements, after sensitivity analysis).

Assuming hyperelastic behaviour, the balloon material derived from the uniaxial tensile test was implemented using a third order Ogden energy function:

$$U = \mathop \sum \limits_{i = 1}^{N} \frac{2\mu }{{\alpha_{i}^{2} }}\left( {\bar{\lambda }_{1}^{{\alpha_{i} }} + \bar{\lambda }_{2}^{{\alpha_{i} }} + \bar{\lambda }_{3}^{{\alpha_{i} }} - 3} \right) + \mathop \sum \limits_{i = 1}^{N} \frac{1}{{\delta_{i} }}(J^{el} - 1)^{2i}$$

where $\bar{\lambda }_{i} = J^{ - 1/3} \lambda_{i }$ are the deviatoric principal stretches, $\lambda_{i }$ are the principal stretches, N is a material parameter, μ_i_, α_i_ and δ_i_ and are temperature-dependent material parameters (Table 2) and J^el^ is the elastic volume ratio. The Poisson’s coefficient was set equal to 0.45 [27].

**Table 2:** Coefficients of the third order Ogden strain energy function used to model the material of the valvuloplasty balloon

Valvuloplasty balloon			
i	μ_i_	α_i_	δ_i_
1	−4715.57	4.89	1.38
2	2480.94	6.90	0
3	2479.35	1.13	0

The experimental conditions were replicated by exploiting Abaqus fluid exchange and fluid cavity capabilities. The inflation flow rate of the in vitro test was imposed as a volumetric flux entering the balloon from the catheter inlet, while the inner balloon pressure *P* and the diameter *d* of the central section were requested as output variables. The experimental boundary conditions were replicated by constraining the extremities of the balloon in the radial directions.

The results were assessed by computing the root-mean-square error ($RMSE_{P} ,\;RMSE_{d}$) between computational and experimental data for pressure and diameter at equal volume.

$$RMSE_{P} = \sqrt {mean\left( {\left( {P_{EXP} - P_{COMP} } \right)^{2} } \right)} ,\;\;\;\;\;\;\;\;RMSE_{d} = \sqrt {mean\left( {\left( {d_{EXP} - d_{COMP} } \right)^{2} } \right)}$$

### Validation of simplified implantation site FE model

The simplified implantation site model was designed as a hollow cylinder (*d* = 14.6 mm, *t* = 1.2 mm, *l* = 60 mm) and meshed with not-reduced hexahedral elements (~60,000 elements, after sensitivity analysis). The adopted material (T50) was modelled as linear, elastic and isotropic, with the same *E* derived from uniaxial tensile test and Poisson’s coefficient equal to 0.25 [28]. The terminal regions of the cylinder (10.6 mm long) were constrained to simulate the experimental set-up of the compliance test. The same internal pressure measured during the experiment was applied to the inner surface of the cylinder. Such model was validated against in vitro results comparing the final internal volume, at the same pressure.

### Validation of the interaction between balloon and simplified implantation site

In order to validate the behaviour of the valvuloplasty balloon inflated into the simplified anatomical site (cylinder with *d* = 22 mm, *t* = 1.2 mm, *l* = 75 mm), mesh and material constitutive law were set identical as per when the two parts were modelled separately. A deflated geometry was firstly obtained by crimping and deflating the balloon in a preliminary simulation, to fit the balloon inside the cylinder. Once positioned, the deflated balloon was virtually inflated by fluid exchange with the same flow rate of the corresponding experimental test. A master–slave, general contact algorithm was defined to allow the interaction between the balloon external surface and the cylinder cavity. *P*–*V* and *d*–*V* curves were obtained as output and compared against the experimental curves for validation purposes.

## Results

### Experimental results

#### Balloon characterisation

**Fig. 3:**
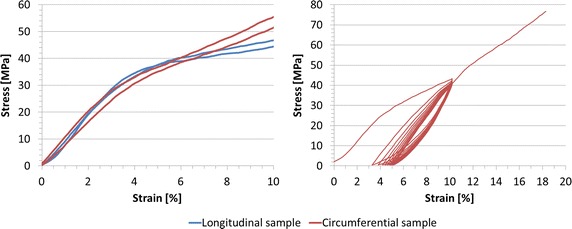
Stress-strain curves of valvuloplasty balloon material. In the *left panel*, data from failure tensile test on circumferential and longitudinal dumb-bell specimens, suggesting the assumption of isotropic behaviour up to 10 % of strain. In the *right panel*, data from cyclic tensile test on circumferential dumb-bell specimens show elastoplastic material behaviour

Stress–strain relationships from the uniaxial tensile tests are shown in Fig. 3. The stress–strain curves suggested that a hyperelastic model was appropriate to describe the material behaviour. Results showed that the hypothesis of isotropic behaviour can be considered valid within the range of deformations of interest (ε < 10 %).

After the first loading cycle (Fig. 3), the specimen exhibited a residual strain of 3 %, which did not increase substantially in the following cycles (3 ÷ 5 %). The presence of hysteresis showing residual strain between loading and unloading curve is a clear manifestation of the elastoplasticity of the material.

#### Inflation tests of balloon

**Fig. 4:**
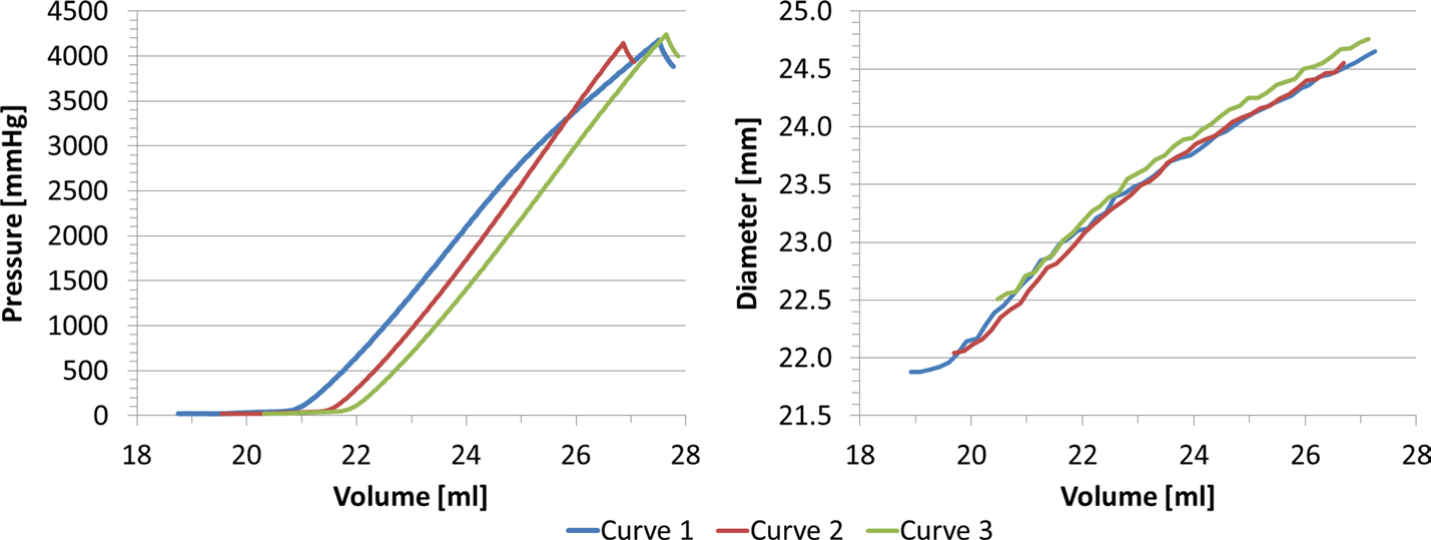
Experimental *P*–*V* and *d*–*V* relationships for valvuloplasty balloon free inflation. In *curve* 1, pressure starts increasing in correspondence of the nominal volume and diameter as reported in the device specifications (*V*_*0*_ = 21 ml and *d*_*0*_ = 22.7 mm); *curves* 2 and 3 show a progressive increment of the nominal values of both volume and diameter (*V*_*0,2*_ = 21.6 ml, *d*_*0,2*_ = 22.9 mm and *V*_*0,3*_ = 22.0 ml, *d*_*0,3*_ = 23.2 mm)

*P*–*V* and *d*–*V* curves from the inflation tests are shown in Fig. 4. Volume varied between 18.7 and 27.8 ml. The resulting *P* and *d* ranges were 0 ÷ 4242 mmHg and 21.9 ÷ 27.3 mm. In agreement with the specification of the valvuloplasty balloon, during the first inflation (curve 1) we observed an increase from the zero pressure in correspondence of the nominal volume of *V*_*0*_ = 21 ml and nominal diameter *d*_*0*_ = 22.7 mm, while a progressive increment of the nominal volume, and consequently of the diameter, was noticed in the following curves 2 (*V*_*0*_ = 21.6 ml, *d*_*0*_ = 22.9 mm) and 3 (*V*_*0*_ = 22.0 ml, *d*_*0*_ = 23.2 mm).

#### Rapid prototyping material characterisation

**Fig. 5:**
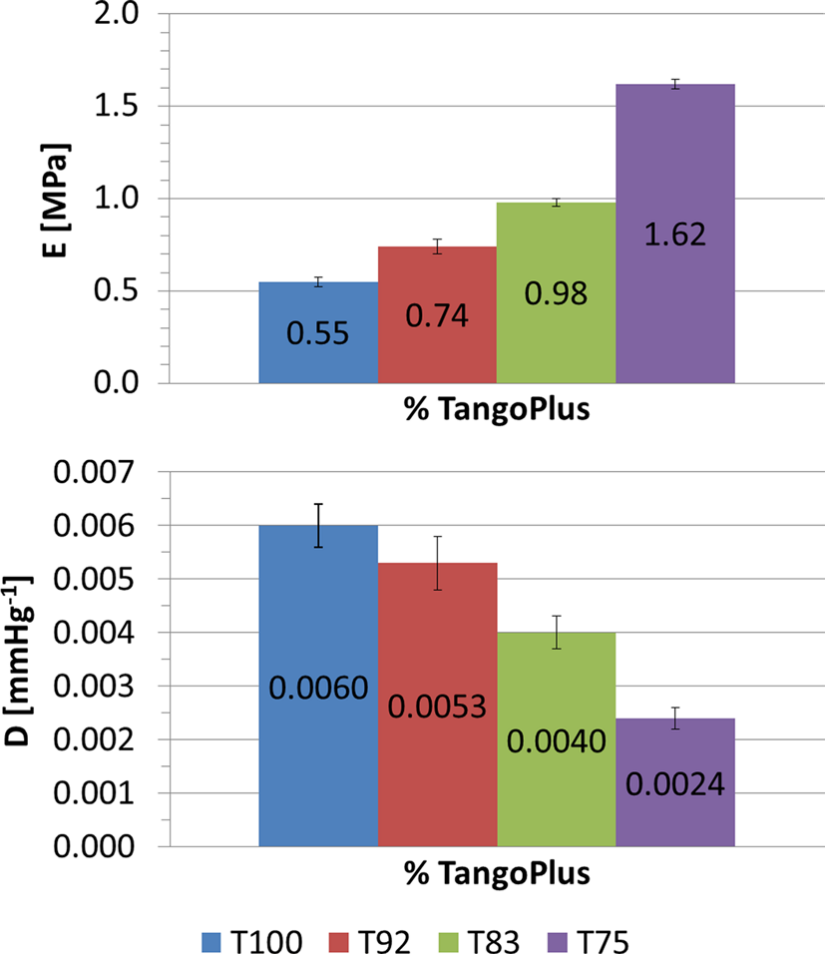
Distensibility and Young’s Modulus obtained from compliance tests on cylinders printed in four different composites (T100, T92, T83, T75). An increase in the percentage of VeroWhite indicates a higher Young’s Modulus and a lower distensibility

**Fig. 6:**
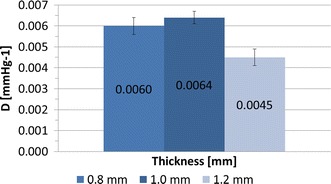
Distensibility obtained from compliance tests on T100 cylinders printed in three different thicknesses (*t* = 0.8 mm, *t* = 1.0 mm, *t* = 1.2 mm). An increase in thickness suggests a moderate decrease in sample distensibility

The composite samples which underwent compliance test exhibited *D* varying in the range 0.0024 ÷ 0.0060 mmHg^−1^, and lower for larger percentages of VeroWhite, as expected (Fig. 5). *E* obtained from compliance tests was ranging from 0.55 MPa (T100) to 1.62 MPa (T75) (Fig. 5). The effect of cylinder thickness on *D* is displayed in Fig. 6 for samples printed in T100, showing a moderate increase in *D* (0.0060 ÷ 0.0045 mmHg^−1^) as the thickness decreases.

The model of simplified implantation site was printed as a 1.2 mm thick cylinder with the material T50, exhibiting *D* = 0.00025 mmHg^−1^ from compliance test and *E* = 13.19 MPa from uniaxial tensile test. The reasons behind such choices were: a) thicknesses of 0.8 and 1.0 mm were found unsuitable to perform inflation experiments, exhibiting high rate of failure (i.e., model rupture following cracking) during the compliance experiments; b) given the lower limit on the thickness and the results of the RP material characterisation, the best solution to mimic *D* (influenced by both thickness and material) and *E* (influenced only by the material) of calcific aorta [24–26] was obtained by increasing to 50 % the percentage of VeroWhite of the composite.

#### Inflation test of balloon inside a simplified implantation site

**Fig. 7:**
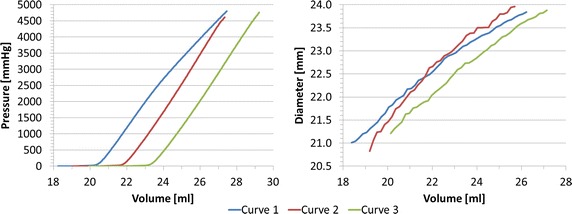
Experimental *P*–*V* and *d*–*V* relationships for valvuloplasty balloon inflated inside the simplified implantation site, showing an increase of the nominal volume and diameter between consecutive inflations

Figure 7 shows *P*–*V* and *d*–*V* curves for the inflation test inside the simplified implantation site. *V* varied between 18.2 and 29.2 ml. The resulting *P* and *d* ranges were 0 ÷ 4801 mmHg and 20.8 ÷ 24.0 mm. As for the inflation of the balloon alone, we observe an increase of the nominal balloon volume (*V*_*0*_) between the three tests (curve 2: *V*_*0*_ = 21.8 ml, *d*_*0*_ = 22.6 mm; curve 3: *V*_*0*_ = 23.3 ml, *d*_*0*_ = 22.6 mm).

### Computational results

The hypothesis of quasi-static condition was verified for all the implemented computational models.

In clinical practice, before delivery, the valvuloplasty balloon undergoes a de-bubbling process to remove residual air, which consists in several unfolding and re-folding of the balloon with contrast medium, with no significant increase in pressure. Therefore, for the valvuloplasty FE balloon, the second curve from the free inflation test was selected as the most representative to validate the behaviour of the balloon in both the tested conditions i.e., free inflation and inflation inside the cylindrical phantom.

#### Validation of balloon FE model

**Fig. 8:**
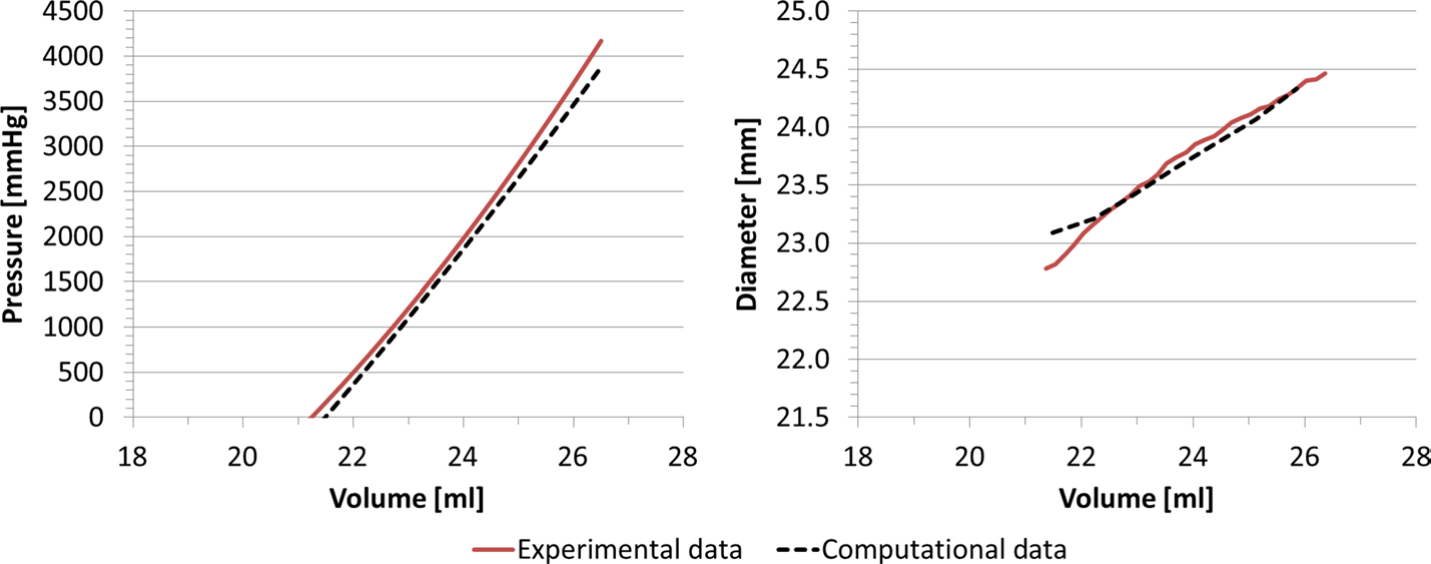
Experimental and computational *P*–*V* and *d*–*V* relationships for valvuloplasty balloon free inflation, leading to *RMSE*_*P*_ = 161.98 mmHg and *RMSE*_*d*_ = 0.12 mm

The agreement between computational and experimental data in terms of *P*–*V* and *d*–*V* curves was good, with *RMSE*_*P*_ = 161.98 mmHg, corresponding to the 3.8 % of the maximum pressure, and *RMSE*_*d*_ = 0.12 mm, inferior to fluoroscopy images resolution (0.5 mm), thus validating the geometry of the valvuloplasty balloon FE model (Fig. 8).

#### Validation of cylinders FE model

Comparing computational results of cylinder deformations with the experimental counterpart yielded an average error on the final volume of 2.82 % (9.97 vs 10.26 ml). The computational model of the cylinder can thus be considered validated to model more complex (i.e., realistic) geometries and interaction with the balloon.

#### Validation of the interaction between balloon and simplified implantation site

**Fig. 9:**
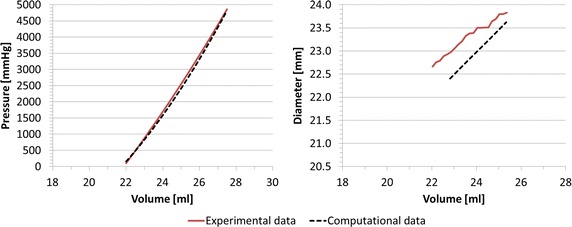
Experimental and computational *P*–*V* and *d*–*V* relationships for valvuloplasty balloon inflated inside the simplified implantation site, leading to *RMSE*_*P*_ = 94.87 mmHg and *RMSE*_*d*_ = 0.49 mm

The numerical simulation of the balloon-in-cylinder system (Fig. 9) showed a good agreement between in vitro and in silico results, with *RMSE*_*P*_ = 94.87 mmHg (1.9 % of the maximum pressure) and *RMSE*_*d*_ = 0.49 mm during the inflation process. The coupling of balloon and cylinder FE model validated the interaction algorithm between the two structures.

## Discussion

This study presents a methodology to characterise non-compliant valvuloplasty balloon behaviour and to validate numerical models of the balloon itself, and of the balloon interacting with an idealised implantation site with realistic mechanical properties.

Balloon geometry and material properties gathered with experimental tests allowed to accurately design a computational model of the device. FE simulations were then able to successfully replicate the experimental data in both inflation scenarios (i.e., balloon alone and balloon within phantom). Such a good agreement was achieved partly due to the accurate design of the device in terms of geometry and material properties, and partly due to the adoption of the fluid cavity and fluid exchange algorithms, which allow to realistically simulating the entrance of a fluid with specified physical properties (i.e., density, bulk modulus) into a closed cavity. Indeed, in order for numerical models to be translated into clinically relevant settings, they need to be robustly validated, which in this case was demonstrated by the good agreement between computational and experimental data. The observed discrepancy between simulated values of pressure and diameter is in fact within the precision characterising the same measurements in the clinical setting. In particular, during ballooning procedures balloon pressure is measured with a manometer incorporated in the inflation system, characterised by full scale range = 0 ÷ 5 atm and resolution = 0.2 atm, and balloon diameter is assessed with fluoroscopy images (isotropic image resolution = 0.5 mm/pixel). Our models exhibit maximum *RMSE*_*P*_ = 161.98 mmHg, comparable to clinical manometer resolution, and maximum *RMSE*_*d*_ = 0.49 mm, lower than fluoroscopy image resolution.

Considering the potential translation of such a validated computational model, there are three clinically relevant scenarios that warrant exploration.

(i) In the case of TAVR, the first step of the procedure involves performing a valvuloplasty i.e., opening the calcified aortic valve, thus creating the suitable landing zone for the replacement valve. If computational models were to be translated in an effort toward personalised healthcare, i.e., providing the ability of performing patient-specific simulations for improving decision-making and tailoring device selection, then the possibility of relying on a validated computational model of the non-compliant balloon would allow to reliably simulate the valvuloplasty procedure, thereby generating the patient-specific implantation site for subsequent virtual TAVR device implantation. This would, however, also require accurate knowledge of calcium and tissue properties in order to carry out realistic simulations of dislodgement of the calcifications and opening of the valve leaflets. This point warrants further investigation.
(ii) Also relevant for TAVR simulations for tailoring device selection, there is a need for a validated numerical model of the balloon in order to carry out virtual stent expansion; indeed literature has discussed how using a displacement algorithm or simplified/idealised balloon models is a sub-optimal solution [29], while having access to a more realistic balloon model would also ensure that the whole virtual device implantation is more reliable when virtually testing balloon-expandable TAVR devices.
(iii) Non-compliant balloons are also used clinically for interventional repair of aortic coarctation and/or other stenosed vessels (e.g., stenosed pulmonary arteries). In these cases, the balloons may be deployed in complex geometries (e.g., aortic arch, vessels bifurcation or tortuosity) which cannot be simplified as a straight tube, as in the presented case of the LVOT. In order for the balloon model to be used in these configurations, a further step would be required to extend the current model, i.e., accounting for the balloon straightening effect on a not-straight implantation site [30]. Such a model would allow performing patient-specific simulations of ballooning procedures, thus providing data on immediate post-interventional vessel configuration, pressure drop across the repaired narrowing, and stresses acting on the vessel wall during the procedure itself. All these information would carry clinical significance.

It should be noted that the validation framework discussed in this paper could easily be generalised to model and validate other devices (e.g., balloons with different size, shape or constitutive material, as indeed different balloons are manufactured by several makers and routinely used in different clinical centres), ultimately leading to more reliable computational simulations in the above-mentioned clinically relevant scenarios of minimally invasive cardiac interventions.

With regards to the valvuloplasty balloon material, this was characterised in both longitudinal and circumferential directions with uniaxial tensile failure tests, showing that the material can be considered isotropic within the working deformation experienced by the balloon (<10 %); the hyperelastic theory was deemed suitable to computationally model this behaviour. Cyclic tests suggested elastoplastic properties for the balloon material.

Free balloon inflation tests were carried out three times on the same balloon. *P*–*V* and *d*–*V* results after the first full inflation highlighted the presence of a residual strain due to the previous stretching of the balloon over its elastic limit. In support of the observation regarding elastoplastic behaviour of the balloon material, we noticed that the increase in diameter after the first inflation was comparable with the ranges of residual strain observed in the cyclic test (Fig. 3). Moreover, we hypothesize that also the rubber-like behaviour of the cylinder may play a role in this process. However, cyclic experimental data on the RP material to confirm such assumption is not available. These findings should be further investigated by repeating the same tests on more than one balloon sample, in order to evaluate experimental repeatability. This limitation does not impinge, however, on the good agreement achieved between computational and experimental data for the purpose of validating the numerical model.

*E* of the RP composite T50 was computed assuming linear elastic isotropic behaviour. Although this hypothesis cannot be fully verified by a rubber-like material like T50, a value of *E* approximating the material behaviour was considered relevant in order to compare the simplified implantation site properties with the available literature data [25, 26] describing calcified arteries mechanical properties in terms of Young’s Modulus. With this regard, we underline the lack of adequate and consistent literature reporting physiological and pathological values of human aortic root material properties.

Although suitable to replicate global properties of certain pathological tissues (i.e., *D* and *E* of calcified aortic root), the RP composites herein adopted present some limitations. In fact, although the PolyJet™ technology specification would in theory allow samples with as low as 0.6 mm thickness, this in practice cannot be suitable for our scope. Indeed we have noticed experimentally a high rate of material cracking and failure for sample thicknesses <1 mm, hence the inevitable decision of printing the implantation site simplified model with a thickness of 1.2 mm to ensure robustness. Moreover, one can assume that this limitation would increase for higher percentage of VeroWhite, due to the brittle component of the material. It would be difficult to replicate the properties of calcified arteries with even higher *E* (i.e., incrementing the percentage of VeroWhite), mainly due to an excessive increase of fragility and reduction of distensibility. In conclusion, the RP materials characterisation suggested that, even mixing different percentage of TangoPlus, responsible for the composite distensibility, and VeroWhite, contributing to its stiffness, only a limited value of the calcific aorta *E* range can be reproduced.

In this work, experimental data were collected for the purpose of designing and validating the numerical model of a specific valvuloplasty balloon. Albeit thorough, the modelling process required some assumptions. The thickness of the balloon, which is not even along the longitudinal direction due to the manufacturing process, was set as uniform. The unfolded configuration of the balloon, obtained from a preliminary deflation simulation, may be improved. Despite these limitations, the model recapitulated satisfactorily the experimental data and this work represents a framework for validating any potential balloon behaviour against specifically gathered experimental tests, provided the knowledge of few, essential device specifications. Moreover, the study highlights the necessity of validating balloon models prior to implementing more complicated scenarios, such as patient-specific simulations.

## Conclusion

In this work, we designed and validated a FE model of a valvuloplasty balloon and its interaction with a simplified cylindrical implantation site, identifying the best combination of different mixtures of RP materials to reproduce the mechanical properties of stiffened arteries/LVOTs. This work represents an additional improvement towards computational modelling of ballooning procedures, in particular for performing virtual valvuloplasty interventions and virtual device implantations for minimally invasive catheterisation, such as TAVR.
